# Gut Microbiota in Autophagy Regulation: New Therapeutic Perspective in Neurodegeneration

**DOI:** 10.3390/life13040957

**Published:** 2023-04-06

**Authors:** Sarmistha Mitra, Yeasmin Akter Munni, Raju Dash, Toma Sadhu, Largess Barua, Md. Ariful Islam, Dipannita Chowdhury, Debpriya Bhattacharjee, Kishor Mazumder, Il Soo Moon

**Affiliations:** 1 Department of Anatomy, College of Medicine, Dongguk University, Gyeongju 38066, Republic of Korea; 2 Department of New Biology, Daegu Gyeongbuk Institute of Science and Technology, Daegu 42988, Republic of Korea; 3 Department of Bioinformatics and Biotechnology, Asian University for Women, Chittagong 4000, Bangladesh; 4 Department of Anatomy and Neurobiology, School of Dentistry, Kyungpook National University, Daegu 41940, Republic of Korea; 5 Department of Pharmaceutical Sciences, North South University, Dhaka 1229, Bangladesh; 6 Department of Pharmacy, BGC Trust University Bangladesh, Chittagong 4381, Bangladesh; 7 Faculty of Environment and Natural Sciences, Brandenburg Technical University Cottbus Senftenberg, D-03013 Cottbus, Germany; 8 Department of Pharmacy, Jashore University of Science and Technology, Jashore 7408, Bangladesh; 9 School of Optometry and Vision Science, UNSW Medicine, University of New South Wales (UNSW), Sydney, NSW 2052, Australia

**Keywords:** gut microbiota, autophagy, neurodegenerative diseases, brain injuries

## Abstract

Gut microbiota and the brain are related via a complex bidirectional interconnective network. Thus, intestinal homeostasis is a crucial factor for the brain, as it can control the environment of the central nervous system and play a significant role in disease progression. The link between neuropsychological behavior or neurodegeneration and gut dysbiosis is well established, but many involved pathways remain unknown. Accumulating studies showed that metabolites derived from gut microbiota are involved in the autophagy activation of various organs, including the brain, one of the major pathways of the protein clearance system that is essential for protein aggregate clearance. On the other hand, some metabolites are evidenced to disrupt the autophagy process, which can be a modulator of neurodegeneration. However, the detailed mechanism of autophagy regulation by gut microbiota remains elusive, and little research only focused on that. Here we tried to evaluate the crosstalk between gut microbiota metabolites and impaired autophagy of the central nervous system in neurodegeneration and the key to future research regarding gut dysbiosis and compromised autophagy in neurodegenerative diseases.

## 1. Introduction

Residing inside the intestine, diverse microorganisms, such as bacteria, archaea, fungi, and viruses, collectively defined as gut microbiota (GM), regulate various cellular functions and host homeostasis [1]. The dynamics of GM start from birth onward and are significant for multiple metabolism-related pathways, especially in regulating brain activity [2]. GM can be considered a vital metabolic organ that plays an essential role in neural development, behavior, cognition, and mood and can modulate neuronal diseases [3,4]. This is because of a bidirectional connection between GM and the central nervous system (CNS), which is established through a neural, enzymatic, and immune pathway called the gut–brain axis (GBA) [5,6]. It is emphasized that altering the GM composition by different therapeutic implications remarkably affects CNS [7]. However, this mutualistic crosstalk between the gut and CNS can be disrupted by a harmful change in the GM composition called gut dysbiosis (GD) [8]. Evidence from clinical and pre-clinical studies concluded that GD is related to neuropsychic and neurodegenerative diseases (NDDs) such as multiple sclerosis (MS) [9], amyotrophic lateral sclerosis diseases (ALS) [10], Parkinson’s disease (PD) [11], Alzheimer’s disease (AD) [12], and Huntington’s disease (HD) [13], to name a few. Mechanistically, GD interrupts the balance of GM-mediated inflammatory and pro-inflammatory processes, inhibits the release of anti-inflammatory cytokines, causes oxidative imbalance, and ultimately disrupts cellular proteostasis function. 

In many age-related diseases, including NDDs, the function of proteostasis has been compromised by the over-accumulation of misfolded protein and protein aggregates due to proteasome failure, the central cellular protein degradation system. In such cases, misfolded protein aggregates can be cleared by autophagy, an intracellular lysosomal catabolic process causing the degradation of unnecessary cytoplasmic macromolecules and restoring cellular energy through the biosynthesis of components [14,15,16]. As the accumulation of toxic misfolded protein is one of the main hallmarks of NDDs [17], disruption of the autophagy may increase misfolded protein aggregates in the brain and promote the occurrences of various types of neurodegenerative disorders [8,18,19]. In addition to the role of GD in promoting inflammation and oxidative imbalance, GD also inhibits the protein degradation capacity by directly regulating autophagy, whereas a healthy gut reversely shows this effect. However, the mechanism by which GM regulates autophagy is still poorly understood. This review summarizes the relationship between GD and NDDs, focusing mainly on autophagy regulation and potential therapeutic interventions to restore intestinal homeostasis and autophagy activation to NDDs. 

In addition, followed by GBA, pro-inflammatory cytokines and lipopolysaccharides (LPSs) innervate permeabilization into the intestine and blood–brain barrier (BBB) from the peripheral nervous system to CNS facilitates the accumulation of misfolded protein [8,20]. The consequences of misfolded protein aggregates can cause neuronal death and neurodegeneration [20]. Numerous in vivo studies suggested that gut microbiota-derived metabolites, including neurotransmitters, neuromodulator peptides, short-chain fatty acids (SCFAs), and LPSs, influence attenuating neuroinflammation [21,22]. Studies have reported that gut metabolites are vital in regulating neurodegenerative and neuroinflammatory disorders [5,23,24] by initiating autophagy [25,26]. 

Besides these, gut metabolites showed a variety of functions in autoimmune, metabolic, and neurodegenerative disorders [27]. Additionally, SCFAs derived from microbiota strongly modulate the pathology of neuronal dysfunction. Gut metabolites upregulate the autophagy flux through GBA and have a direct link with several CNS pathways where autophagy function reduces the spreading of the neuro-inflammatory mediator and contributes to attenuating the progressive neurodegeneration by eliminating the cellular debris [28]. Several studies suggested that various factors, including endoplasmic reticulum stress, oxidative stress, and aging, impair the function of autophagy and play a role in the development of numerous neurodegenerative disorders, including AD, PD, and HD [29]. Moreover, gut metabolites control the permeability of toxic substances into BBB and convey protection from developing oxidative stress [25,30,31]. Moreover, gut metabolites upregulate the multiple pathways associated with autophagy in the epithelial layer and reduce gut barrier injury [32]. Multiple clinical and pre-clinical experiments showed that probiotic supplementation balances GD [27] and thus improves oxidative stress conditions by influencing autophagy [33]. In this review, we summarize the relationship between the dysbiosis of GM and NDDs by regulating autophagy and potential therapeutic intervention to restore intestinal homeostasis.

## 2. Gut Microbiota (GM) and Brain Relationship

The bidirectional relationship between intestinal microbiota and the brain is a well-known phenomenon through which various microorganisms can play a role in cognition, neuro-physiological behavior, and diseases [34]. GM maintains the gut–brain relationship via endocrinal, immune, anatomical, and neural pathways [35,36,37]. The neuroanatomical pathways between the gut and brain include the autonomic nervous system (ANS) and vagus nerve (VN) in the spinal cord, and communication between gut and brain through the enteric nervous system (ENS) in the gut and ANS and VN within the spinal cord [38]. The neuroendocrine system is the hypothalamic–pituitary–adrenal (HPA) axis, which functions in response to stress and chemical and mechanical stimuli of GM [34]. GM and brain connection mechanisms also include the production of bacterial metabolites and various immune mediators, such as cytokines, and direct signaling via VN [39,40]. VN can modulate the activity of enteric neurons to communicate with CNS [41]. Gut bacteria influence these central processes through their ability to synthesize neurotransmitters (i.e., γ-amino butyric acid (GABA), noradrenaline, and dopamine) [42], which subsequently influence microglial activation and cerebral function [43]. Moreover, GM modulates the activation of the immune system, along with its ability to produce metabolites, such as SCFAs, that possess neuroactive properties [39]. SCFAs are the main metabolites produced by bacterial fermentation of dietary fibers [44], including propionic acid, butyric acid, and acetic acid, which are responsible for modulating various signaling and exerting neuroactive properties [31]. Many studies suggested that SCFAs may directly interact to regulate brain function, as they are reportedly present in cerebrospinal fluid (CSF) [45].

Bidirectional gut–brain communication is also an essential player in the mechanism of neurodegeneration, as this stimulates the brain for several significant functions, such as neurotransmission of signals, neurogenesis, neuroinflammation, and activation of the stress axes, together with modulating behaviors [46]. Surprisingly, this relationship depends on the microbial community in the individual host, so balancing good and harmful microbe populations is crucial. While the GM composition is critical in CNS regulation and disease progression, different behavioral disruptions also control the GM composition [47]. Thus, the optimum balance between beneficial and harmful microbes is defined as “homeostasis”, and the disruption of this balance is called “dysbiosis” [48].

GD has been considered one of the most important causative factors of neurodegeneration [49], which is recognized by the increase of harmful microbes. The hostile bacteria include *Enterobacteriaceae*, consisting of gut commensals *Escherichia*, *Shigella*, *Proteus*, and *Klebsiella*. Increasing these harmful microorganisms release large amounts of harmful metabolites, causing increased permeability of intestinal barriers. Consequently, GD increases systematic inflammation with chronic inflammatory diseases and other metabolic diseases [49]. Again, GD is linked with impaired autophagy and decreased levels of autophagy-related proteins, such as microtubule-associated protein light chain 3 (LC3) [50,51]. Reportedly, intestinal dysbiosis is linked with the disturbance of autophagy; on the other hand, continual autophagy activation is mandatory for the brain to function correctly [52]. Thus, a proper balance of autophagy is required for neuronal development and CNS function. The mutual relationship between intestinal microbiota in controlling autophagy can be targeted as a potential therapeutic approach for NDDs.

## 3. Autophagy and Neurodegeneration

“Autophagy” is derived from a Greek word meaning “self-eating”, an evolutionarily conserved process that degrades and recycles cellular components [53]. It can also be defined as a multistep intracellular degradation process by the formation of a double-membrane autophagosome [54,55], which is engulfed by cytoplasm for the bulk degradation of intracellular waste materials, such as damaged organelles [56], misfolded/aggregated proteins [57,58], and intracellular pathogens (e.g., bacteria, fungi, or viruses) [59]. In mammalian cells, three basic types of autophagy have been identified: macroautophagy, chaperon-mediated autophagy (CMA), and microautophagy [57]. Again, macroautophagy is defined as “selective autophagy” that can exclusively sequester and degrade protein aggregates [60]. Macroautophagy is an essential quality control system, which is part of basal constitutive autophagy induced by different stressors, such as protein aggregation and proteasome failure [61]. Major NDDs have been linked to the accumulation of abnormal protein aggregation in neurons, glial cells, and the extracellular space, such as β-amyloid peptide (Aβ) plaques and tau-positive neurofibrillary tangles (NFTs) in AD [62]; α-synuclein-positive Lewy bodies in PD [63]; tau [64], TDP-43- [65], and FUS-positive aggregates in frontotemporal dementia [66]; and aggregates of a mutant form of huntingtin (HTT) in HD [67]. Thus, autophagy is a neuroprotective mechanism to degrade aggregate-prone cytoplasmic proteins that cause these NDDs [68,69,70,71,72,73]. 

Several selective autophagy receptors have been identified that interact with the cargo and components of the autophagic machinery, thus providing a molecular basis for selective degradation [68]. Yeast genetics has been vital for elucidating the molecular machinery involved in autophagy processes [74]. So far, 34 autophagy-related (ATG) genes have been reported in yeast, and 15 of these are “core” ATG genes commonly required for the different autophagy pathways [75]. Autophagy initiates when the mammalian target of rapamycin complex 1 (mTORC1) is inhibited, activating the Ulk1-Atg13-FIP200 complex, which triggers Beclin1, Bcl-2 family proteins, class III phosphatidylinositol 3-kinase (Vps34), and the Atg14l complex to initiate autophagosome formation [76]. Following Beclin1 activation, the Atg5-Atg12 conjugation system and the microtubule-associated protein light-chain 3 (LC3-Atg8) conjugation system regulate the elongation of autophagosome [77]. In the cytoplasm, autophagosomes develop randomly and are carried by microtubules to the microtubule organizing center. Following that, lysosomal acid proteases destroy the contents of the autophagosome, and the degradation products are released for metabolic recycling [78]. 

As the autophagy pathway is involved in NDDs’ causative misfolded protein, a defective autophagy pathway is directly linked to neurodegeneration [69,70,71,72,73,79,80,81,82,83]. In PD, increased alpha-synuclein levels inhibit autophagy by mislocalizing ATG9, a protein with critical functions in autophagosome formation [84]. In HD, the mutant version of huntingtin with expanded polyQ repeats forms toxic protein aggregates that affect the autophagy pathway at various steps. The autophagy protein BECN1 was found to be reduced in the hippocampus of schizophrenia patients [85]. Moreover, some features of autism spectrum disorder, such as social behavior defects or repetitive behavior, are triggered by impaired microglial autophagy [86]. The deletion of the autophagy gene Atg7 in microglia was associated with autistic behavior in mice models [87]. 

Inducing autophagy by overexpression of autophagy genes or proteins can be essential in reducing neurodegeneration as a therapeutic approach. One study reported that brain-derived neurotrophic factors could provide neuroprotection from hypoxia by inducing autophagy via the PI3K/Akt/mTOR/p70S6K signaling pathway [88]. In a rat model of subarachnoid hemorrhage, autophagy activation by rapamycin or inhibitor 3-methyladenine is connected to neuroprotection against apoptosis through a mitochondrial route [89]. As aggregate-prone proteins are highly dependent on the autophagy pathway for clearance, inducing autophagy can be a potential therapeutic option for treating proteinopathies [90].

## 4. Relation of Gut Microbiota (GM) and Autophagy in Neurodegeneration 

There is a smooth balance between GM and autophagy that is regulated bidirectionally. This relationship has recently been in the limelight in many studies because its imbalance is associated with many disease progressions. While the roles of GM and its metabolites in autophagy are excessively studied and highlighted in intestinal homeostasis, few studies focus only on the brain [26,91]. However, our discussion is more directed at explaining the observed and possible mechanisms of GM-mediated regulation of autophagy and vice versa. 

Among various NDDs, the relationship between GD and PD is widely studied because GBA is directly involved in transporting misfolding of alpha-synuclein from the gut to the brain at the early stages of pathology [92,93,94,95,96]. The most popular method to study the pathological behavior of PD is using MPTP (1-methyl-4-phenyl-1,2,3,6-tetrahydropyridine), which is a neurotoxic-to-dopaminergic neuron once it is metabolized to 1-methyl-4-phenylpyridine (MPP+) [97]. Since MPTP can damage dopaminergic neurons in ENS, it is considered a reliable model for studying GM relations in PD. A recent study shows that MPTP triggered GD and intestinal pathology by changing microbes’ composition before motor function failure, and chronic administration with a low dosage of MPTP is suitable for GM-related studies [98]. Rotenone is another example that has been demonstrated to imitate the clinical and pathological features of PD quite well [99,100], and it is also closely related to GD when PD is induced by chronic administration [101,102,103]. Using an MPTP (1-methyl-4-phenyl-1,2,3,6-tetrahydropyridine)-based mice PD model, Liu et al. identified the autophagy interplay between gut and brain concerning GD and PD. They observed that chronic administration of MPTP changes GM composition and alters gut autophagy, and it is also associated with reduced propionate, acetate, and SCFAs [104]. The observation of this study concludes that metabolites that are secreted from the GM are responsible for autophagy regulation. Indeed, the treatment of sodium butyrate in an in vitro PD model (rotenone-treated PC12 cells) induced autophagy by increasing PGC-1α expression [105]. Moreover, sodium butyrate is reported to reduce α-synuclein clearance through activating autophagy-mediated clearance by regulating PI3K/Akt/mTOR-related and Atg5-dependent pathways in enteroendocrine cells [106]. Li et al. discovered that metabolites produced by microbes in the gut are responsible for the induction of mitophagy, reducing microglia-mediated neuroinflammation [107]. 

Accumulating studies other than NDDs provide strong evidence of autophagy failure caused by GD. For example, Gu et al. reported that GD, induced by bisphenol F, was associated with autophagy reduction and neuroinflammation in zebrafish brains [108]. Unlike MPTP and rotenone, bisphenol F does not directly affect ENS but changes microbial composition by increasing potential pathogenic bacteria [109]. Conversely, the treatment of autophagy inducers was also reported to reshape GM for exerting protective effects against neurotoxicity. For example, in experimental autoimmune encephalomyelitis (EAE)-based MS animal model, the treatment of MCC950 and an autophagy activator, rapamycin, recovered GD in normal mice and slowed down disease progression by inducing autophagy [110]. However, in many cases, GM recovery from GD can also inhibit autophagy for a neuroprotective effect. For example, the remodeling of GM by fecal microbiome transplantation (FMT), which was disturbed by Mn exposure, a neurotoxic condition resembling PD, showed neuroprotection by inhibiting autophagy in the hippocampus through the regulating apelin signaling pathway [111]. Similarly, autophagy inhibition by disrupting beclin1 heterozygous remodels GM from GD, which is induced by arsenite, alleviates neurobehavioral impairments via gut–brain communication [112]. Martin et al. showed that, in the presence of infectious and non-infectious intestinal risks, autophagy proteins inhibit a beneficial microbiota-induced type I interferon (IFN-I) response, suggesting that proteins involved in autophagy regulate immune response to the brain at the gut barrier [113].

**Figure 1:**
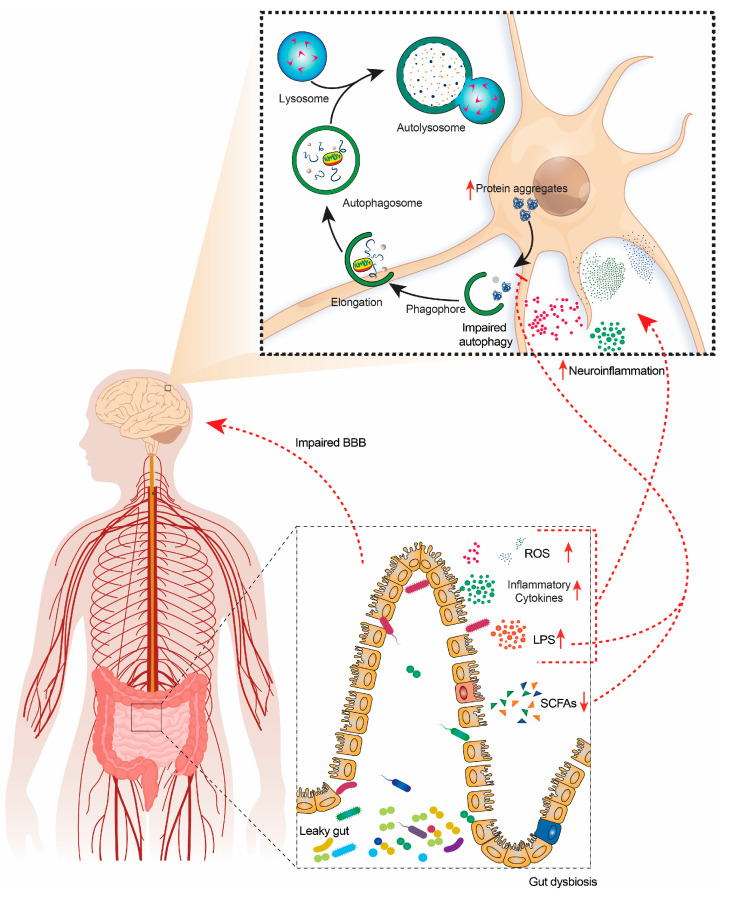
Graphical representation of the involvement of GM-derived metabolites in regulating autophagy and brain degeneration. Gut dysbiosis (GD) increases the production of harmful metabolites, such as LPS and inflammatory cytokines, and significantly reduces beneficial metabolites, such as SCFA. GD reduces the integrity of the intestinal epithelial barrier and BBB by releasing harmful metabolites into systematic circulation and the brain. Increased inflammatory mediators cause neuroinflammation, leading to protein aggregation and neurodegeneration. Moreover, due to increased LPS and reduced SCFA, autophagy is disrupted, hindering the clearance of toxic protein aggregates. LPSs, lipopolysaccharides; SCFAs, short chain fatty acids; BBB, blood–brain barrier; ROS, reactive oxygen species.

Other factors, such as diet and GM-targeted therapies, have also been reported to modulate autophagy in various disease conditions. Wang et al. observed significant changes in the GM of offspring when mother mice were fed high-sugar and high-fat diets for one month and found an increased level of autophagy in the brain with an elevated expression of different LC3 levels [114]. In the aging mice model, supplementation with Urolithin A, derived from the GM through the biotransformation of ellagitannins, induced autophagy through the miR-34a-mediated inhibition of the mTOR signaling pathway and upregulating SIRT1 [115]. Using 3xTgAD mice, Bonfili et al. showed that alterations in the microbiome reduce neuronal proteolysis, but when supplemented with probiotics (SLAB51, composed of nine live bacterial strains), they improved the degradation mechanism of Aβ42 by promoting the ubiquitin–proteasome function [116]. The authors also identified that SLAB51 administration promotes autophagy-mediated clearance by regulating the SIRT1 pathway [117]. Changes in gut flora observed in AD may affect autophagic flux and, thus, the clearance system in the brain, as shown by a few studies that demonstrated that restoring GM via prebiotic treatment triggers autophagy by the PI3K/Akt/mTOR pathway [118]. More detailed studies are required to elucidate the interaction between brain autophagy and GM, especially in neurodegenerative conditions. A good balance between GM composition and autophagy regulation is perhaps essential for proper host homeostasis. A summary of the autophagy regulation by GM or vice versa is illustrated in Figure 1 and Table 1. 

## 5. Role of Gut Microbiota (GM) and Metabolites in NDD Pathogenesis

Numerous studies in humans and experimental animals have pointed to the role of microbiota in the onset and progression of NDDs accomplished by various microbial metabolites via the GBA or CNS [6,95,96,97]. As stated in earlier sections, different gut microbial metabolites consist of neuromodulators, anti-inflammatory, pro-inflammatory agents, and uremic toxins [90,91,92,93,94] that have been linked to various physiological systems of the host’s body together with neural development and the maintenance of brain function, and also maintaining the integrity of BBB [22]. This section discusses the role of microbes and metabolites regulating the pathogenesis of common NDDs, such as AD, PD, HD, MS, and ALS. 

### 5.1. Parkinson’s Disease (PD)

The misfolding and aggregation of α-synuclein in damaged brain areas containing cytoplasmic clumps called Lewy bodies (LBs) are the clinical outcomes of PD that are responsible for neuronal loss and degeneration [119,120,121]. Motor impairment is observed in PD because of the gradual loss of dopaminergic neurons in the midbrain’s Substantial Nigra [122]. PD affects around one percent of persons over 65 worldwide; a small percentage suffer from familial PD or parkinsonism [123]. Nevertheless, in addition to these causes, several studies have revealed that PD pathology may develop predominantly through the gut [124,125,126,127]. Nearly eighty percent of people diagnosed with PD experience GI symptoms before experiencing motor signs [125]. Faecalibacterium, Lachnospiraceae, and Prevotellaceae were found in the patients’ feces at lower levels than in controls [128], suggesting GI problems associated with PD and thus inspiring researchers to investigate the connections between gut microorganisms and the disease. 

GM change may affect PD since the mesentery (spleen, pancreas, and GI tract) contributes approximately half of the body’s dopamine (DA) production [129]. Altschuler et al. postulated in 1996 that *Helicobacter pylori* (*H. pylori*) infection had a causal role in the pathophysiology of PD [130]. Higher Lactobacillaceae and lower Prevotellaceae may also modify nigrostriatal dopamine activity and slow PD progression by decreasing gut hormones such as ghrelin [131,132]. The connection between GD and alterations in microbial metabolites in PD was also extensively explored. SCFAs are the metabolic by-products of GM activities, including propionate, butyrate, and acetate. SCFAs are being investigated as molecular therapies for reverse NDDs because they tend to regulate the interconnections between the GM and the CNS [31]. PD patients were shown to have lower levels of SCFA-generating microbiota, such as Prevotellaceae and Lachnospiraceae, according to the investigations that were conducted in the past [133,134]. Moreover, the SCFAs acetate, butyrate, and propionate were also found in PD patients’ feces in lower concentrations than in age-matched controls [135,136]. Urolithin A (UA) is a metabolite produced when the digestive-tract bacteria act to convert ellagitannins into a different component. Oxidative processes caused by MAO-A can be prevented by using the antioxidant UA, which is suggested as a possible metabolite for treating NDDs [137] since ROS (reactive oxygen species) production has been established as a critical factor in NDDs. UA promotes mitochondrial biogenesis through SIRT1-PGC-1α signaling, resulting in neuroprotection in 6-OHDA-induced PC-12 cells. Both 6-OHDA-induced motor impairments and nigral–striatal dopaminergic neurotoxicity were attenuated when UA was administered to the PD mice [138]. Kujawska et al. showed protection against oxidative damage and α-synuclein aggregation in the PD rat model by administering pomegranate juice where the concentration of UA was 1.68 ± 0.25 ng/g tissue and in plasma 18.75 ± 3.21 ng/mL in the brain [139]. Trimethylamine-N-oxide, also known as TMAO, is a metabolite that is produced by the GM and found in high amounts in seafood, dairy products, red meats, muscle, and egg yolks [140,141]. TMAO has been reported to induce proper protein folding [142] and lessen the production of the aggregation of α-synuclein fibrils [142]. Studies indicate that people with PD had lower levels of TMAO, increasing dementia associated with levodopa-equivalent doses [143]. A disruption in the gut barrier causes the translocation of bacterial components such as LPS from the intestine into the bloodstream of PD patients. As a result, the Toll-like receptor 4 (TLR4)-mediated pathway is activated, promoting inflammation and exacerbating PD neurodegeneration [144]. In animal models of PD, increased levels of Tryptophan metabolites and KYNA in the brain were found to protect nigrostriatal dopamine neurons against QUIN-induced excitotoxin damage [145]. Additionally, vitamins are necessary minerals that humans can obtain only through diet or microbiota in their digestive tract, where vitamin K is derived from the bacteria Escherichia coli, Klebsiella pneumonia, Eubacterium, and Propionibacterium [146]. It has been demonstrated that vitamin K plays a favorable function as an anti-fibrillogenic on α-synuclein aggregation, which is directly related to PD [147]. In summary, consistent evidence suggests that the microbiota in the gut and the microbial metabolites produced by those microbes have a major influence on the modulation of PD.

### 5.2. Alzheimer’s Disease (AD)

AD is the most common form of dementia, and its proportion is rising as the world’s population ages [148]; it is characterized by the accumulation of β-amyloid peptide (Aβ) and a microtubule-associated protein known as tau [149]. Prior to the onset of AD progression, numerous studies in vivo have documented shifts in microbial communities (*Rikenellaceae*, *Erysipelotrichaceae*, *Bacteroidaceae*, *Verrucomicrobiaceae*, *Wolbachia*, *Rikenellaceae*, *Prevotellaceae*, *Proteobacteriaceae*, and *Bifidobacteriaceae*) before any plaque formation in the brain [150], suggesting the involvement of GM in the pathogenesis of AD. Studying the brain function of 8-month-old transgenic (Tg) mice of AD, Gu et al. discovered that Tg mice exhibited lower amounts of SCFA-producing bacteria (such as *Parasutterella* and *Blautia*) and more GD compared to wild-type mice [151]. Notably, there have been a number of recent investigations in which proteins (bacterial, viral, or fungal-derived) and nucleic acid have been found in the brains of AD patients who had already passed away, indicating that microbial metabolites such as SCFAs may be generated locally [152,153]. The SCFAs propionic, butyric, and isobutyric acids were reduced in AD-model mice compared to wild-type mice [154,155,156]. In contrast, increasing butyrate intake through food supplements has been proven to improve AD pathogenies by boosting memory-related genomic programs and re-establishing DNA acetylation [157]. Butyrate was proven to increase both the survival rate and the motor ability in a dose-dependent manner of R6/2 transgenic mice in a model of HD [158]. Previous studies have identified that SCFAs can prevent Aβ aggregation in vitro [159]. In APP/PS1 transgenic mice, treatment with UA improved cognitive function, reduced neuronal death, and increased neurogenesis [160]. UA can induce autophagy to increase Aβ clearance in neuronal cell lines [161]. Ali et al. provided evidence that anthocyanin, derived from microbial metabolism, modulates p-PI3K/Akt/GSK3β pathways, decreasing amyloid beta oligomer in both in vitro and in vivo models of a model of AD (APP/PS1) [162]. Though limited, new data suggest that anthocyanins may also exert neuroprotective effects by directly preventing protein aggregation and stimulating autophagy, as concluded by Aimee et al. [163]. The expression and activity of Neprilysin are increased by tryptophan metabolites, 5-hydroxy indole-acetic acid (5-HIAA), and kynurenic acid (KYNA), which stimulates the elimination of Aβ in the brain-protecting neurons from Aβ-peptide-induced toxicity [164]. There are several types of metabolites, such as phytosphingosine, dihydrosphingosine, hypoxanthine, and inosine, that have been shown to reduce the symptoms of AD where the administration of xanthoceraside, a molecule with anti-activity of AD, was associated with changes in the levels of these metabolites and a shift in the composition of gut bacterial taxonomy, suggesting novel avenues for treating AD. [165]. It is known that microtubule disassembly and neuronal death are the hallmark pathogenic aspects of AD [166], and TMAO has been reported to rescue the capacity of mutant tau to induce microtubule assembly [167,168]. Like PD, vitamins play an essential role in the modulation of AD [169]. High doses of B vitamins, such as B6, B9, and B12, have been shown to reduce levels of homocysteine, a by-product of vitamin B, and prevent the degeneration of some brain regions linked to cognitive impairment in AD [170].

### 5.3. Huntington’s Disease (HD)

HD is a hereditary autosomal-dominant neurodegenerative illness that is caused by the mutation of the huntingtin (Htt) protein, with an expanded polyglutamine (polyQ) stretch leading to Htt fragments and the formation of aggregates [171,172]. The gut may also play a role in moderating the pathogenesis of HD because its microbiota is disrupted before severe cognitive and motor impairments are set in [173]. Massive changes in the structure of bacterial communities at the phylum and family levels and impacted metabolic systems and enzymes were also discovered in our HD patients [13]. The existence of GD was verified by a 16 s RNA-sequencing genomic profile of the GM from a fecal sample of transgenic HD mice [174], where 16 s rRNA sequencing of R6/1 HD mice showed a sex-specific GM composition. According to a recent clinical study, specific GM components, including *actinobacteria*, are significantly more prevalent in HD patients than in healthy participants by DNA extraction after feces and blood samples had been collected for analysis [175]. In addition, the gut permeability of R6/2 mice with HD was enhanced, and the microbiome was significantly altered. More specifically, Bacteroidetes (Gram^-^) were more abundant than Firmicutes (Gram^+^) in these animals [176]. Additionally, KYNA plays a vital role in HD. HD patients exhibit abnormal TRP metabolism and increased oxidative stress. These factors contribute to continued brain dysfunction. Plasma levels of KYNA, 3-HK, and 3-HAA and KAT activity are decreased in HD [177]. GM produces ellagic acid (EA). EA dramatically decreased mHTT levels, neuroinflammation, and oxidative stress, preventing further synapse loss in R6/2 mice [178]. All of these findings point to a link between NDDs and GD, and they emphasize the importance of a balanced microbiota composition in preventing the development of HD.

### 5.4. Amyotrophic Lateral Sclerosis (ALS)

ALS is a deadly neurological disease that mainly affects adults. It is diagnosed by the selective loss of motor neurons. ALS is also known as familial ALS or fALS since it can be passed down from parent to child in around 10% of all instances [179]. Compared to controls, ALS patients showed lower butyrate-producing bacteria, such as *Eubacterium rectale* and *Roseburia intestinalis* [180], which regulate gut integrity and inflammation [181]. Another study showed that a high production of SCFAs is associated with a high body mass index (BMI) [182] in people; on the other hand, a high BMI is known to be inversely correlated with ALS risks [183,184]. Moreover, the administration of *Akkermansia muciniphila* caused an increase of nicotinamide in the CNS, alleviating various symptoms of ALS, enhancing motor symptoms, and modifying the expression of genes in SOD1-Tg mice [185]. One study showed no statistically significant changes between patient and healthy groups in the levels of KYNA in serum or CSF. Despite this, the concentration of KYNA in the CSF was shown to be significantly greater in patients with severe clinical problems compared to healthy control subjects, and there was no correlation between the KYNA concentrations in the serum and CSF [186]. Consistent with the observations of several experiments, microbiome-targeted metabolite therapies might be potential future routes for preventing ALS due to the multiple connections between ALS and GM. 

### 5.5. Multiple Sclerosis (MS)

Demyelination and axonal degeneration of the CNS are hallmarks of the autoimmune disease known as MS [187]. Patients with MS were shown to have abnormalities in the gut flora associated with feces. *Caulobacteraceae*, *Pseudomonas*, and *Mycoplana* were found in higher abundance in MS patients, but *Enterobacteriales* were found in higher abundance in the healthy controls [188]. Furthermore, in animals hyper-sensitive to a myelin oligodendrocyte glycoprotein, continuous administration of colistin, kanamycin, and vancomycin suppressed experimental autoimmune encephalomyelitis (EAE) progression, but in V14 natural killer T (iNKT)-cell-deficient mice, this activity was lost [189]. The SCFA propionic acid (PA) induces T cells expressions in the gut, which, in turn, has a beneficial effect on the CNS in MS patients [190]. I3S and other aryl hydrocarbon receptor (AHR) agonists can be generated by symbiotic-bacteria-producing tryptophan metabolites, reducing the harmful activity of astrocytes in MS mice [191]. Reduced numbers of *Parabacteroides*, *Revotella*, and *Adlercreutzia* are associated with a higher risk of multiple sclerosis, even though these bacteria can maintain mucosal surface homeostasis, generate anti-inflammatory effects, and serve as phytoestrogen metabolite producers in MS. [188]. In another study, untreated MS patients were found to have lower levels of *Collinsella* and *Slackia*, both of which belong to the phyla Actinobacteria and Prevotella. In contrast, when comparing treated MS patients to untreated MS patients, the authors found that *Prevotella* and *Sutterella* levels were higher in the treated MS patients [192]. The above findings suggest that the GM population has a major impact on the progression of autoimmune illnesses such as MS and that GM manipulation should be considered a therapeutic option in treating MS in the coming decades.

In addition, gut patients with MS who received tryptophan metabolites improved their cognitive and memory functions [193,194,195]. The expression of AhR on microglia and astrocytes is boosted by tryptophan metabolites (I3S, tryptamine, indole-3-acetic acid, kynurenine, and kynurenic acid), which also play a role in the regulation of inflammatory processes [196,197]. Since GPBAR1 expresses in glial and immune cells, secondary metabolites, such as bile acid can modulate the reactivity of astrocytes [198]. The SCFAs propionic acid (PA) raises the number of regulatory T cells originating from the gut, and this benefits the CNS in MS patients [190]. As a whole, various metabolites in the digestive tract can prevent the progression of multiple sclerosis.

## 6. Therapeutic Interventions and Future Perspectives

**Figure 2:**
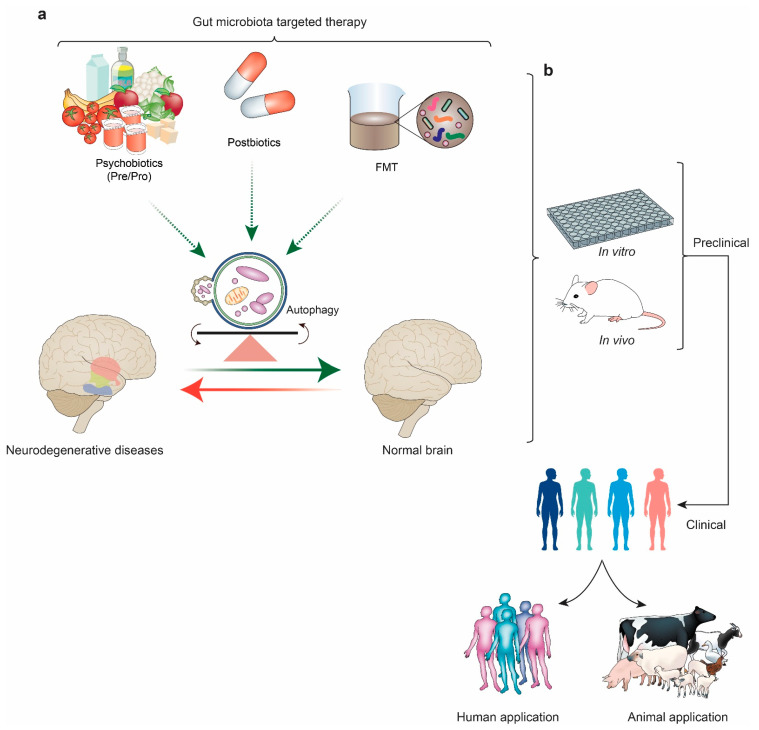
Illustration representing the therapeutic application and future research potential of gut microbiota (GM)-derived autophagy regulation in the modulation of neurodegenerations. (**a**) Showing psychobiotics (prebiotics and/or probiotics), postbiotics, and FMT as a therapeutic method to modulate GM. Positive modulation of GM can either initiate or inhibit autophagy and its association with neurodegeneration, a potential aspect that needs extensive mechanistic studies in the future. (**b**) Representing the future research methodologies using GM to establish proper therapeutics. In vitro and in vivo research and clinical trials for proper therapeutic application (in humans and animals) are the main focus of future research regarding GM. FMT, fecal microbiota transplant.

Introducing beneficial GM and re-establishing the balance can be a fruitful method to correct dysbiosis, and, as a result, impaired autophagy can be restored. Many external factors modify the gut community of microbiota. Diet, exercise, lifestyle, environment, stress, etc., are the various factors that affect gut microbial development. The most well-known methods of therapeutic application may include postbiotics and psychobiotics [199], FMT, and diet modification (Figure 2). Psychobiotics are defined as a combination of “prebiotics” and “probiotics” that can especially affect the gut–brain relationship [36]. Probiotics are helpful bacteria, and prebiotics is compounds that promote helpful bacteria growth. Psychobiotics treatments are known to improve neurological and behavioral conditions, as established by many studies [200,201]. Postbiotics include any metabolites or products that the GM produces that can positively or indirectly affect the host [202]. Cell-free supernatant from GM, enzymes, cell fragments, SCFA, and bacterial lysates are especially considered for therapeutic application. Besides restoring intestinal-balance postbiotics, these products help correct immune system disruption [203]. Bacterial peptidoglycan, considered postbiotic treatment, is also responsible for autophagy induction in epithelial cells [204] and the liver [205]. Again, healthy eating and diet play an important role in establishing the balance of GM [206]. Surprisingly, diet modification by caloric restriction can help in autophagy induction [207] and neuropsychiatric conditions [208]. Furthermore, in a recent study, it was proved that the high sugar and high fat (HSHF) diet of a mother amends the GM of the offspring and the expression of neuronal and autophagy markers in the brain during the early life stage [114]. If aspects of the microbiome are easily modifiable, for example, it may be possible to alter autophagic flux through dietary modifications. Foods that promote SCFA formation via microbial fermentation in the colon, such as those prevalent in the Mediterranean diet, reduce the signs of frailty and cognitive impairment in the elderly by altering the richness and character of the intestinal microbiota [209]. FMT has shown great promise to repair the GD that leads to NDDs. To treat dysbiosis, a healthy donor’s feces containing gut bacteria can be delivered to the patient by using an enema or nasogastric, nasoenteric, or endoscopic methods [210].

The relationship between GM and the brain is well-studied and evident in numerous in vivo and in vitro studies. In this review, we highlighted the brain and GM relationship and focused on the autophagic mechanism that can be one mechanistic perspective of the relationship. Autophagy is a crucial player in NDDs’ progression. Until now, the involvement of GD has been reported in the autophagy of various organs, especially in the intestinal epithelial barrier [211], heart [212], muscles [213], and liver [51]. SCFA is reported to induce hepatic autophagy in a UCP2-dependent pathway, and the loss of the gut microbiome can impair basal liver autophagy [51]. This review also discussed the possible mechanisms of GM autophagy in CNS and the bidirectional relationship between GM autophagy and nervous system disorder. Thus, GM-mediated autophagy can be a potential mediator in NDD treatment. To understand the mechanism of GM and its metabolites in nervous system autophagy, a further mechanistic study must be performed. Studies in animal models of NDD and human patients must be performed in future research to establish the relationship and possible therapeutic approach. The evidence linking the compromised autophagy of CNS and GM dysbiosis is compelling; however, clinical and pre-clinical studies need more attention for therapeutic application.

## Figures and Tables

**Table 1:** Summary of studies that highlighted the reciprocal relationship between gut homeostasis and brain autophagy.

Study Model	Study Type	Modulators		Mechanism	Reference
		Disease/GD	Autophagy		
Parkinson’s disease	In vivo	MPTP (Neurotoxic model for PD)		Changes GM composition Alters gut autophagy Reduces propionate, acetate, and SCFAs	[104]
Parkinson’s disease	In vitro (PC12 cell)	Rotenone (Neurotoxic model for PD)	Sodium butyrate	Sodium butyrate induced autophagy by increasing PGC-1α expression	[105]
Multiple sclerosis	In vivo (Experimental autoimmune encephalomyelitis)		MCC950 and Rapamycin	Slowed down disease progression Improved GD and recovered GM composition	[110]
Alzheimer’s disease	In vivo (3× Tg mice)		Probiotics (SLAB51)	SLAB51 induces autophagy and proteolysis Improve GM composition Activate SIRT1 pathway	[117]
Alzheimer’s disease	In vivo (3× Tg mice)		Probiotics (SLAB51)	Partial restoration of ubiquitin proteasome system and autophagy Promote degradation of Aβ42	[116]
Ageing mice model/oxidative damage model on cell	In vivo/in vitro (PC12 cell)		Urolithin A	Induces autophagy by inhibiting mTOR pathway Upregulates SIRT1	[115]
Parkinson’s disease	In vivo	Manganese-induced neurotoxicity resembles PD	Fecal microbiota transplant (FMT)	Provides neuroprotection Inhibits autophagy by the apelin signaling pathway.	[111]
Parkinson’s disease	In vitro (Enteroendocrine cells)		Sodium butyrate	Reduces α-synuclein clearance by activating autophagy Activates PI3K/Akt/mTOR-related pathway Activates Atg5-dependent pathways	[106]
Neurological disorder mice model	In vivo		Microbial metabolite NAMO	Restores NAD+-dependent mitophagy Inhibit microglia activation and HSE progression	[107]
Alzheimer’s disease rodent model	In vivo	Aβ_1-42_-induced rats	Fructooligosaccharide	Improves memory by triggering autophagy Activates PI3K/Akt/mTOR pathway	[118]

## Data Availability

Not applicable.

## References

[1] Arumugam M., Raes J., Pelletier E., Le Paslier D., Yamada T., Mende D.R., Fernandes G.R., Tap J., Bruls T., Batto J.M. (2011). Enterotypes of the human gut microbiome. Nature.

[2] Derrien M., Alvarez A.-S., de Vos W.M. (2019). The Gut Microbiota in the First Decade of Life. Trends Microbiol..

[3] Moloney E.B., de Winter F., Verhaagen J. (2014). ALS as a distal axonopathy: Molecular mechanisms affecting neuromuscular junction stability in the presymptomatic stages of the disease. Front. Neurosci..

[4] Guinane C.M., Cotter P.D. (2013). Role of the gut microbiota in health and chronic gastrointestinal disease: Understanding a hidden metabolic organ. Ther. Adv. Gastroenterol..

[5] Silva Y.P., Bernardi A., Frozza R.L. (2020). The Role of Short-Chain Fatty Acids From Gut Microbiota in Gut-Brain Communication. Front. Endocrinol..

[6] Zhang H., Chen Y., Wang Z., Xie G., Liu M., Yuan B., Chai H., Wang W., Cheng P. (2022). Implications of Gut Microbiota in Neurodegenerative Diseases. Front. Immunol..

[7] Ávila P.R.M., Fiorot M., Michels M., Dominguini D., Abatti M., Vieira A., de Moura A.B., Behenck J.P., Borba L.A., Botelho M.E.M. (2020). Effects of microbiota transplantation and the role of the vagus nerve in gut–brain axis in animals subjected to chronic mild stress. J. Affect. Disord..

[8] Chidambaram S.B., Essa M.M., Rathipriya A.G., Bishir M., Ray B., Mahalakshmi A.M., Tousif A.H., Sakharkar M.K., Kashyap R.S., Friedland R.P. (2022). Gut dysbiosis, defective autophagy and altered immune responses in neurodegenerative diseases: Tales of a vicious cycle. Pharmacol. Ther..

[9] Zeng Q., Junli G., Liu X., Chen C., Sun X., Li H., Zhou Y., Cui C., Wang Y., Yang Y. (2019). Gut dysbiosis and lack of short chain fatty acids in a Chinese cohort of patients with multiple sclerosis. Neurochem. Int..

[10] Wu S., Yi J., Zhang Y.-g., Zhou J., Sun J. (2015). Leaky intestine and impaired microbiome in an amyotrophic lateral sclerosis mouse model. Physiol. Rep..

[11] Wang Q., Luo Y., Ray Chaudhuri K., Reynolds R., Tan E.-K., Pettersson S. (2021). The role of gut dysbiosis in Parkinson’s disease: Mechanistic insights and therapeutic options. Brain.

[12] Liu S., Gao J., Zhu M., Liu K., Zhang H.-L. (2020). Gut Microbiota and Dysbiosis in Alzheimer’s Disease: Implications for Pathogenesis and Treatment. Mol. Neurobiol..

[13] Wasser C.I., Mercieca E.-C., Kong G., Hannan A.J., McKeown S.J., Glikmann-Johnston Y., Stout J.C. (2020). Gut dysbiosis in Huntington’s disease: Associations among gut microbiota, cognitive performance and clinical outcomes. Brain Commun..

[14] Ohsumi Y. (2014). Historical landmarks of autophagy research. Cell Res..

[15] Glick D., Barth S., Macleod K.F. (2010). Autophagy: Cellular and molecular mechanisms. J. Pathol..

[16] Ma X., Lu C., Chen Y., Li S., Ma N., Tao X., Li Y., Wang J., Zhou M., Yan Y.B. (2022). CCT2 is an aggrephagy receptor for clearance of solid protein aggregates. Cell.

[17] Bourdenx M., Koulakiotis N.S., Sanoudou D., Bezard E., Dehay B., Tsarbopoulos A. (2017). Protein aggregation and neurodegeneration in prototypical neurodegenerative diseases: Examples of amyloidopathies, tauopathies and synucleinopathies. Prog. Neurobiol..

[18] Aziz M.N.M., Kumar J., Muhammad Nawawi K.N., Raja Ali R.A., Mokhtar N.M. (2021). Irritable Bowel Syndrome, Depression, and Neurodegeneration: A Bidirectional Communication from Gut to Brain. Nutrients.

[19] Kesika P., Suganthy N., Sivamaruthi B.S., Chaiyasut C. (2021). Role of gut-brain axis, gut microbial composition, and probiotic intervention in Alzheimer’s disease. Life Sci..

[20] Roy Sarkar S., Banerjee S. (2019). Gut microbiota in neurodegenerative disorders. J. Neuroimmunol..

[21] Russo R., Cristiano C., Avagliano C., De Caro C., La Rana G., Raso G.M., Canani R.B., Meli R., Calignano A. (2018). Gut-brain axis: Role of lipids in the regulation of inflammation, pain and CNS diseases. Curr. Med. Chem..

[22] Ahmed H., Leyrolle Q., Koistinen V., Kärkkäinen O., Layé S., Delzenne N., Hanhineva K. (2022). Microbiota-derived metabolites as drivers of gut-brain communication. Gut Microbes.

[23] Rogers G.B., Keating D.J., Young R.L., Wong M.L., Licinio J., Wesselingh S. (2016). From gut dysbiosis to altered brain function and mental illness: Mechanisms and pathways. Mol. Psychiatry.

[24] Franceschi C., Garagnani P., Parini P., Giuliani C., Santoro A. (2018). Inflammaging: A new immune-metabolic viewpoint for age-related diseases. Nat. Rev. Endocrinol..

[25] Wu M., Luo Q., Nie R., Yang X., Tang Z., Chen H. (2021). Potential implications of polyphenols on aging considering oxidative stress, inflammation, autophagy, and gut microbiota. Crit. Rev. Food Sci. Nutr..

[26] Lapaquette P., Bizeau J.B., Acar N., Bringer M.A. (2021). Reciprocal interactions between gut microbiota and autophagy. World J. Gastroenterol..

[27] Castelli V., d’Angelo M., Quintiliani M., Benedetti E., Cifone M.G., Cimini A. (2021). The emerging role of probiotics in neurodegenerative diseases: New hope for Parkinson’s disease?. Neural Regen. Res..

[28] Shoubridge A.P., Fourrier C., Choo J.M., Proud C.G., Sargeant T.J., Rogers G.B. (2021). Gut Microbiome Regulation of Autophagic Flux and Neurodegenerative Disease Risks. Front. Microbiol..

[29] Yin Y., Sun G., Li E., Kiselyov K., Sun D. (2017). ER stress and impaired autophagy flux in neuronal degeneration and brain injury. Ageing Res. Rev..

[30] Hoyles L., Snelling T., Umlai U.-K., Nicholson J.K., Carding S.R., Glen R.C., McArthur S. (2018). Microbiome–host systems interactions: Protective effects of propionate upon the blood–brain barrier. Microbiome.

[31] Dalile B., Van Oudenhove L., Vervliet B., Verbeke K. (2019). The role of short-chain fatty acids in microbiota–gut–brain communication. Nat. Rev. Gastroenterol. Hepatol..

[32] Cheng S., Ma X., Geng S., Jiang X., Li Y., Hu L., Li J., Wang Y., Han X. (2018). Fecal microbiota transplantation beneficially regulates intestinal mucosal autophagy and alleviates gut barrier injury. Msystems.

[33] Wu Y., Wang B., Xu H., Tang L., Li Y., Gong L., Wang Y., Li W. (2019). Probiotic Bacillus attenuates oxidative stress-induced intestinal injury via p38-mediated autophagy. Front. Microbiol..

[34] Mitra S., Dash R., Nishan A.A., Habiba S.U., Moon I.S. (2022). Brain modulation by the gut microbiota: From disease to therapy. J. Adv. Res..

[35] Montiel-Castro A., González-Cervantes R., Bravo-Ruiseco G., Pacheco-Lopez G. (2013). The microbiota-gut-brain axis: Neurobehavioral correlates, health and sociality. Front. Integr. Neurosci..

[36] Cryan J.F., Dinan T.G. (2012). Mind-altering microorganisms: The impact of the gut microbiota on brain and behaviour. Nat. Rev. Neurosci..

[37] Wang H.-X., Wang Y.-P., Chen X. (2016). Gut Microbiota-brain Axis. Chin. Med. J..

[38] Schemann M., Neunlist M. (2004). The human enteric nervous system. Neurogastroenterol. Motil..

[39] Sherwin E., Sandhu K.V., Dinan T.G., Cryan J.F. (2016). May the Force Be With You: The Light and Dark Sides of the Microbiota–Gut–Brain Axis in Neuropsychiatry. CNS Drugs.

[40] Dinan T.G., Stilling R.M., Stanton C., Cryan J.F. (2015). Collective unconscious: How gut microbes shape human behavior. J. Psychiatr. Res..

[41] Browning K.N., Verheijden S., Boeckxstaens G.E. (2017). The Vagus Nerve in Appetite Regulation, Mood, and Intestinal Inflammation. Gastroenterology.

[42] Sherwin E., Dinan T.G., Cryan J.F. (2018). Recent developments in understanding the role of the gut microbiota in brain health and disease. Ann. N. Y. Acad. Sci..

[43] Abdel-Haq R., Schlachetzki J.C.M., Glass C.K., Mazmanian S.K. (2018). Microbiome–microglia connections via the gut–brain axis. J. Exp. Med..

[44] Pascale A., Marchesi N., Marelli C., Coppola A., Luzi L., Govoni S., Giustina A., Gazzaruso C. (2018). Microbiota and metabolic diseases. Endocrine.

[45] Nagashima H., Morio Y., Meshitsuka S., Yamane K., Nanjo Y., Teshima R. (2010). High-resolution nuclear magnetic resonance spectroscopic study of metabolites in the cerebrospinal fluid of patients with cervical myelopathy and lumbar radiculopathy. Eur. Spine J..

[46] Foster J.A., Lyte M., Meyer E., Cryan J.F. (2016). Gut Microbiota and Brain Function: An Evolving Field in Neuroscience. Int. J. Neuropsychopharmacol..

[47] Mu C., Yang Y., Zhu W. (2016). Gut Microbiota: The Brain Peacekeeper. Front. Microbiol..

[48] Das B., Nair G.B. (2019). Homeostasis and dysbiosis of the gut microbiome in health and disease. J. Biosci..

[49] Spielman L.J., Gibson D.L., Klegeris A. (2018). Unhealthy gut, unhealthy brain: The role of the intestinal microbiota in neurodegenerative diseases. Neurochem. Int..

[50] Carino A., Marchianò S., Biagioli M., Scarpelli P., Bordoni M., Di Giorgio C., Roselli R., Fiorucci C., Monti M.C., Distrutti E. (2021). The bile acid activated receptors GPBAR1 and FXR exert antagonistic effects on autophagy. FASEB J..

[51] Iannucci L.F., Sun J., Singh B.K., Zhou J., Kaddai V.A., Lanni A., Yen P.M., Sinha R.A. (2016). Short chain fatty acids induce UCP2-mediated autophagy in hepatic cells. Biochem. Biophys Res. Commun..

[52] Nikoletopoulou V., Papandreou M.E., Tavernarakis N. (2015). Autophagy in the physiology and pathology of the central nervous system. Cell Death Differ..

[53] Cavallucci V., Fidaleo M., Pani G. (2020). Nutrients and neurogenesis: The emerging role of autophagy and gut microbiota. Curr. Opin. Pharmacol..

[54] Galluzzi L., Baehrecke E.H., Ballabio A., Boya P., Bravo-San Pedro J.M., Cecconi F., Choi A.M., Chu C.T., Codogno P., Colombo M.I. (2017). Molecular definitions of autophagy and related processes. Embo J.

[55] Boya P., Reggiori F., Codogno P. (2013). Emerging regulation and functions of autophagy. Nat Cell Biol.

[56] Butsch T.J., Ghosh B., Bohnert K.A. (2021). Organelle-Specific Autophagy in Cellular Aging and Rejuvenation. Adv. Geriatr. Med. Res..

[57] Lamark T., Johansen T. (2012). Aggrephagy: Selective Disposal of Protein Aggregates by Macroautophagy. Int. J. Cell Biol..

[58] Simonsen A., Wollert T. (2022). Don’t forget to be picky—Selective autophagy of protein aggregates in neurodegenerative diseases. Curr. Opin. Cell Biol..

[59] Wileman T. (2013). Autophagy as a defence against intracellular pathogens. Essays Biochem..

[60] Mizushima N., White E., Rubinsztein D.C. (2021). Breakthroughs and bottlenecks in autophagy research. Trends Mol. Med..

[61] Johansen T., Lamark T. (2011). Selective autophagy mediated by autophagic adapter proteins. Autophagy.

[62] Gallardo G., Holtzman D.M., Takashima A., Wolozin B., Buee L. (2019). Amyloid-β and Tau at the Crossroads of Alzheimer’s Disease. Tau Biology.

[63] Recasens A., Dehay B. (2014). Alpha-synuclein spreading in Parkinson’s disease. Front. Neuroanat..

[64] Dani M., Brooks D.J., Edison P. (2016). Tau imaging in neurodegenerative diseases. Eur. J. Nucl. Med. Mol. Imaging.

[65] Lee E.B., Lee V.M.Y., Trojanowski J.Q. (2012). Gains or losses: Molecular mechanisms of TDP43-mediated neurodegeneration. Nat. Rev. Neurosci..

[66] Deng H., Gao K., Jankovic J. (2014). The role of FUS gene variants in neurodegenerative diseases. Nat. Rev. Neurol..

[67] Saudou F., Humbert S. (2016). The Biology of Huntingtin. Neuron.

[68] Conway O., Akpinar H.A., Rogov V.V., Kirkin V. (2020). Selective Autophagy Receptors in Neuronal Health and Disease. J. Mol. Biol..

[69] Dash R., Ali M.C., Jahan I., Munni Y.A., Mitra S., Hannan M.A., Timalsina B., Oktaviani D.F., Choi H.J., Moon I.S. (2021). Emerging potential of cannabidiol in reversing proteinopathies. Ageing Res. Rev..

[70] Dash R., Jahan I., Ali M.C., Mitra S., Munni Y.A., Timalsina B., Hannan M.A., Moon I.S. (2021). Potential roles of natural products in the targeting of proteinopathic neurodegenerative diseases. Neurochem. Int..

[71] Dash R., Mitra S., Ali M.C., Oktaviani D.F., Hannan M.A., Choi S.M., Moon I.S. (2021). Phytosterols: Targeting Neuroinflammation in Neurodegeneration. Curr. Pharm. Des..

[72] Hannan M.A., Rahman M.A., Rahman M.S., Sohag A.A.M., Dash R., Hossain K.S., Farjana M., Uddin M.J. (2020). Intermittent fasting, a possible priming tool for host defense against SARS-CoV-2 infection: Crosstalk among calorie restriction, autophagy and immune response. Immunol. Lett..

[73] Mitra S., Dash R., Munni Y.A., Selsi N.J., Akter N., Uddin M.N., Mazumder K., Moon I.S. (2022). Natural Products Targeting Hsp90 for a Concurrent Strategy in Glioblastoma and Neurodegeneration. Metabolites.

[74] Nakatogawa H., Suzuki K., Kamada Y., Ohsumi Y. (2009). Dynamics and diversity in autophagy mechanisms: Lessons from yeast. Nat. Rev. Mol. Cell Biol..

[75] Lynch-Day M.A., Klionsky D.J. (2010). The Cvt pathway as a model for selective autophagy. FEBS Lett..

[76] Li X., He L., Che K.H., Funderburk S.F., Pan L., Pan N., Zhang M., Yue Z., Zhao Y. (2012). Imperfect interface of Beclin1 coiled-coil domain regulates homodimer and heterodimer formation with Atg14L and UVRAG. Nat. Commun..

[77] Nishida Y., Arakawa S., Fujitani K., Yamaguchi H., Mizuta T., Kanaseki T., Komatsu M., Otsu K., Tsujimoto Y., Shimizu S. (2009). Discovery of Atg5/Atg7-independent alternative macroautophagy. Nature.

[78] Jahreiss L., Menzies F.M., Rubinsztein D.C. (2008). The Itinerary of Autophagosomes: From Peripheral Formation to Kiss-and-Run Fusion with Lysosomes. Traffic.

[79] Hannan M.A., Dash R., Sohag A.A.M., Haque M.N., Moon I.S. (2020). Neuroprotection Against Oxidative Stress: Phytochemicals Targeting TrkB Signaling and the Nrf2-ARE Antioxidant System. Front. Mol. Neurosci..

[80] Hassan S.S.U., Samanta S., Dash R., Karpiński T.M., Habibi E., Sadiq A., Ahmadi A., Bunagu S. (2022). The neuroprotective effects of fisetin, a natural flavonoid in neurodegenerative diseases: Focus on the role of oxidative stress. Front Pharm..

[81] Mitra S., Dash R., Sohel M., Chowdhury A., Munni Y.A., Ali M.C., Hannan M.A., Islam M.T., Moon I.S. (2022). Targeting estrogen signaling in the radiation-induced neurodegeneration: Possible role of phytoestrogens. Curr. Neuropharmacol..

[82] Rahman M.A., Dash R., Sohag A.A.M., Alam M., Rhim H., Ha H., Moon I.S., Uddin M.J., Hannan M.A. (2021). Prospects of Marine Sterols against Pathobiology of Alzheimer’s Disease: Pharmacological Insights and Technological Advances. Mar. Drugs.

[83] Ripon M.K.H., Lee H., Dash R., Choi H.J., Oktaviani D.F., Moon I.S., Haque M.N. (2020). N-acetyl-D-glucosamine kinase binds dynein light chain roadblock 1 and promotes protein aggregate clearance. Cell Death Dis..

[84] Winslow A.R., Chen C.W., Corrochano S., Acevedo-Arozena A., Gordon D.E., Peden A.A., Lichtenberg M., Menzies F.M., Ravikumar B., Imarisio S. (2010). α-Synuclein impairs macroautophagy: Implications for Parkinson’s disease. J Cell Biol..

[85] Merenlender-Wagner A., Malishkevich A., Shemer Z., Udawela M., Gibbons A., Scarr E., Dean B., Levine J., Agam G., Gozes I. (2015). Autophagy has a key role in the pathophysiology of schizophrenia. Mol. Psychiatry.

[86] Kim H.J., Cho M.H., Shim W.H., Kim J.K., Jeon E.Y., Kim D.H., Yoon S.Y. (2017). Deficient autophagy in microglia impairs synaptic pruning and causes social behavioral defects. Mol. Psychiatry.

[87] Sakai M., Yu Z., Hirayama R., Nakasato M., Kikuchi Y., Ono C., Komatsu H., Nakanishi M., Yoshii H., Stellwagen D. (2022). Deficient Autophagy in Microglia Aggravates Repeated Social Defeat Stress-Induced Social Avoidance. Neural Plast..

[88] Chen A., Xiong L.-J., Tong Y., Mao M. (2013). Neuroprotective effect of brain-derived neurotrophic factor mediated by autophagy through the PI3K/Akt/mTOR pathway. Mol. Med. Rep..

[89] Jing C.h., Wang L., Liu P.p., Wu C., Ruan D., Chen G. (2012). Autophagy activation is associated with neuroprotection against apoptosis via a mitochondrial pathway in a rat model of subarachnoid hemorrhage. Neuroscience.

[90] Winslow A.R., Rubinsztein D.C. (2008). Autophagy in neurodegeneration and development. Biochim. Et Biophys. Acta (BBA)—Mol. Basis Dis..

[91] Larabi A., Barnich N., Nguyen H.T.T. (2020). New insights into the interplay between autophagy, gut microbiota and inflammatory responses in IBD. Autophagy.

[92] Elfil M., Kamel S., Kandil M., Koo B.B., Schaefer S.M. (2020). Implications of the Gut Microbiome in Parkinson’s Disease. Mov. Disord..

[93] Toh T.S., Chong C.W., Lim S.-Y., Bowman J., Cirstea M., Lin C.-H., Chen C.-C., Appel-Cresswell S., Finlay B.B., Tan A.H. (2022). Gut microbiome in Parkinson’s disease: New insights from meta-analysis. Park. Relat. Disord..

[94] Boertien J.M., Pereira P.A.B., Aho V.T.E., Scheperjans F. (2019). Increasing Comparability and Utility of Gut Microbiome Studies in Parkinson’s Disease: A Systematic Review. J. Park. Dis..

[95] Pavan S., Prabhu A.N., Prasad Gorthi S., Das B., Mutreja A., Shetty V., Ramamurthy T., Ballal M. (2022). Exploring the multifactorial aspects of Gut Microbiome in Parkinson’s Disease. Folia Microbiol..

[96] Wallen Z.D., Demirkan A., Twa G., Cohen G., Dean M.N., Standaert D.G., Sampson T.R., Payami H. (2022). Metagenomics of Parkinson’s disease implicates the gut microbiome in multiple disease mechanisms. Nat. Commun..

[97] Hisahara S., Shimohama S. (2011). Toxin-Induced and Genetic Animal Models of Parkinson’s Disease. Park. Dis..

[98] Lai F., Jiang R., Xie W., Liu X., Tang Y., Xiao H., Gao J., Jia Y., Bai Q. (2018). Intestinal Pathology and Gut Microbiota Alterations in a Methyl-4-phenyl-1,2,3,6-tetrahydropyridine (MPTP) Mouse Model of Parkinson’s Disease. Neurochem. Res..

[99] Tasselli M., Chaumette T., Paillusson S., Monnet Y., Lafoux A., Huchet-Cadiou C., Aubert P., Hunot S., Derkinderen P., Neunlist M. (2013). Effects of oral administration of rotenone on gastrointestinal functions in mice. Neurogastroenterol. Motil..

[100] Inden M., Kitamura Y., Takeuchi H., Yanagida T., Takata K., Kobayashi Y., Taniguchi T., Yoshimoto K., Kaneko M., Okuma Y. (2007). Neurodegeneration of mouse nigrostriatal dopaminergic system induced by repeated oral administration of rotenone is prevented by 4-phenylbutyrate, a chemical chaperone. J. Neurochem..

[101] Dodiya H.B., Forsyth C.B., Voigt R.M., Engen P.A., Patel J., Shaikh M., Green S.J., Naqib A., Roy A., Kordower J.H. (2020). Chronic stress-induced gut dysfunction exacerbates Parkinson’s disease phenotype and pathology in a rotenone-induced mouse model of Parkinson’s disease. Neurobiol. Dis..

[102] Yang X., Qian Y., Xu S., Song Y., Xiao Q. (2018). Longitudinal Analysis of Fecal Microbiome and Pathologic Processes in a Rotenone Induced Mice Model of Parkinson’s Disease. Front. Aging Neurosci..

[103] Bhattarai Y., Si J., Pu M., Ross O.A., McLean P.J., Till L., Moor W., Grover M., Kandimalla K.K., Margolis K.G. (2021). Role of gut microbiota in regulating gastrointestinal dysfunction and motor symptoms in a mouse model of Parkinson’s disease. Gut Microbes.

[104] Liu X., Du Z.-R., Wang X., Luk K.-H., Chan C.-H., Cao X., Zhao Q., Zhao F., Wong W.-T., Wong K.-H. (2021). Colonic Dopaminergic Neurons Changed Reversely With Those in the Midbrain via Gut Microbiota-Mediated Autophagy in a Chronic Parkinson’s Disease Mice Model. Front. Aging Neurosci..

[105] Zhang Y., Xu S., Qian Y., He X., Mo C., Yang X., Xiao Q. (2022). Sodium butyrate attenuates rotenone-induced toxicity by activation of autophagy through epigenetically regulating PGC-1α expression in PC12 cells. Brain Res..

[106] Qiao C.-M., Sun M.-F., Jia X.-B., Shi Y., Zhang B.-P., Zhou Z.-L., Zhao L.-P., Cui C., Shen Y.-Q. (2020). Sodium butyrate causes α-synuclein degradation by an Atg5-dependent and PI3K/Akt/mTOR-related autophagy pathway. Exp. Cell Res..

[107] Li F., Wang Y., Song X., Wang Z., Jia J., Qing S., Huang L., Wang Y., Wang S., Ren Z. (2022). The intestinal microbial metabolite nicotinamide n-oxide prevents herpes simplex encephalitis via activating mitophagy in microglia. Gut Microbes.

[108] Gu J., Zhu Y., Guo M., Yin X., Liang M., Lou X., Chen J., Zhou L., Fan D., Shi L. (2022). The potential mechanism of BPF-induced neurotoxicity in adult zebrafish: Correlation between untargeted metabolomics and gut microbiota. Sci. Total Environ..

[109] Wang Y., Wang B., Wang Q., Liu Y., Liu X., Wu B., Lu G. (2021). Intestinal toxicity and microbial community disorder induced by bisphenol F and bisphenol S in zebrafish. Chemosphere.

[110] Xu L., Zhang C., He D., Jiang N., Bai Y., Xin Y. (2020). Rapamycin and MCC950 modified gut microbiota in experimental autoimmune encephalomyelitis mouse by brain gut axis. Life Sci..

[111] Liu J., Zhang X., Ta X., Luo M., Chang X., Wang H. (2022). Fecal microbiome transplantation attenuates manganese-induced neurotoxicity through regulation of the apelin signaling pathway by inhibition of autophagy in mouse brain. Ecotoxicol. Environ. Saf..

[112] Zhang Y., Jiang X., Zhang J., Xia Y., Qiu J., Wang T., Qiu Y., Qin X., Wang B., Zou Z. (2020). Heterozygous disruption of beclin 1 mitigates arsenite-induced neurobehavioral deficits via reshaping gut microbiota-brain axis. J. Hazard. Mater..

[113] Martin P.K., Marchiando A., Xu R., Rudensky E., Yeung F., Schuster S.L., Kernbauer E., Cadwell K. (2018). Autophagy proteins suppress protective type I interferon signalling in response to the murine gut microbiota. Nat. Microbiol..

[114] Wang D., Zhang H., Zeng M., Tang X., Zhu X., Guo Y., Qi L., Xie Y., Zhang M., Chen D. (2021). Maternal high sugar and fat diet benefits offspring brain function via targeting on the gut-brain axis. Aging (Albany NY).

[115] Chen P., Chen F., Lei J., Li Q., Zhou B. (2019). Activation of the miR-34a-Mediated SIRT1/mTOR Signaling Pathway by Urolithin A Attenuates D-Galactose-Induced Brain Aging in Mice. Neurotherapeutics.

[116] Bonfili L., Cecarini V., Berardi S., Scarpona S., Suchodolski J.S., Nasuti C., Fiorini D., Boarelli M.C., Rossi G., Eleuteri A.M. (2017). Microbiota modulation counteracts Alzheimer’s disease progression influencing neuronal proteolysis and gut hormones plasma levels. Sci. Rep..

[117] Bonfili L., Cecarini V., Cuccioloni M., Angeletti M., Berardi S., Scarpona S., Rossi G., Eleuteri A.M. (2018). SLAB51 Probiotic Formulation Activates SIRT1 Pathway Promoting Antioxidant and Neuroprotective Effects in an AD Mouse Model. Mol. Neurobiol..

[118] Chen D., Yang X., Yang J., Lai G., Yong T., Tang X., Shuai O., Zhou G., Xie Y., Wu Q. (2017). Prebiotic Effect of Fructooligosaccharides from Morinda officinalis on Alzheimer’s Disease in Rodent Models by Targeting the Microbiota-Gut-Brain Axis. Front. Aging Neurosci..

[119] Wolters E., Braak H. (2006). Parkinson’s disease: Premotor clinico-pathological correlations. J. Neural. Transm. Suppl..

[120] Braak H., Braak E. (2000). Pathoanatomy of Parkinson’s disease. J. Neurol..

[121] Hawkes C.H., Del Tredici K., Braak H. (2007). Parkinson’s disease: A dual-hit hypothesis. Neuropathol. Appl. Neurobiol..

[122] Zhang H., Tong R., Bai L., Shi J., Ouyang L. (2016). Emerging targets and new small molecule therapies in Parkinson’s disease treatment. Bioorg. Med. Chem..

[123] Warren N., O’Gorman C., Lehn A., Siskind D. (2017). Dopamine dysregulation syndrome in Parkinson’s disease: A systematic review of published cases. J. Neurol. Neurosurg. Psychiatry.

[124] Sampson T.R., Debelius J.W., Thron T., Janssen S., Shastri G.G., Ilhan Z.E., Challis C., Schretter C.E., Rocha S., Gradinaru V. (2016). Gut Microbiota Regulate Motor Deficits and Neuroinflammation in a Model of Parkinson’s Disease. Cell.

[125] Su A., Gandhy R., Barlow C., Triadafilopoulos G. (2017). A practical review of gastrointestinal manifestations in Parkinson’s disease. Park. Relat. Disord..

[126] Edwards L.L., Pfeiffer R.F., Quigley E.M., Hofman R., Balluff M. (1991). Gastrointestinal symptoms in Parkinson’s disease. Mov. Disord..

[127] Savica R., Carlin J.M., Grossardt B.R., Bower J.H., Ahlskog J.E., Maraganore D.M., Bharucha A.E., Rocca W.A. (2009). Medical records documentation of constipation preceding Parkinson disease: A case-control study. Neurology.

[128] Scheperjans F., Aho V., Pereira P.A., Koskinen K., Paulin L., Pekkonen E., Haapaniemi E., Kaakkola S., Eerola-Rautio J., Pohja M. (2015). Gut microbiota are related to Parkinson’s disease and clinical phenotype. Mov. Disord..

[129] González-Arancibia C., Urrutia-Piñones J., Illanes-González J., Martinez-Pinto J., Sotomayor-Zárate R., Julio-Pieper M., Bravo J.A. (2019). Do your gut microbes affect your brain dopamine?. Psychopharmacology.

[130] Altschuler E. (1996). Gastric Helicobacter pylori infection as a cause of idiopathic Parkinson disease and non-arteric anterior optic ischemic neuropathy. Med. Hypotheses.

[131] Queipo-Ortuño M.I., Seoane L.M., Murri M., Pardo M., Gomez-Zumaquero J.M., Cardona F., Casanueva F., Tinahones F.J. (2013). Gut microbiota composition in male rat models under different nutritional status and physical activity and its association with serum leptin and ghrelin levels. PLoS ONE.

[132] Chang R.C.-C. (2011). Advanced Understanding of Neurodegenerative Diseases.

[133] Minato T., Maeda T., Fujisawa Y., Tsuji H., Nomoto K., Ohno K., Hirayama M. (2017). Progression of Parkinson’s disease is associated with gut dysbiosis: Two-year follow-up study. PLoS ONE.

[134] Tagliabue A., Elli M. (2013). The role of gut microbiota in human obesity: Recent findings and future perspectives. Nutr. Metab. Cardiovasc. Dis..

[135] Unger M.M., Spiegel J., Dillmann K.U., Grundmann D., Philippeit H., Bürmann J., Faßbender K., Schwiertz A., Schäfer K.H. (2016). Short chain fatty acids and gut microbiota differ between patients with Parkinson’s disease and age-matched controls. Park. Relat. Disord..

[136] Bedarf J.R., Hildebrand F., Coelho L.P., Sunagawa S., Bahram M., Goeser F., Bork P., Wüllner U. (2017). Functional implications of microbial and viral gut metagenome changes in early stage L-DOPA-naïve Parkinson’s disease patients. Genome Med..

[137] Cásedas G., Les F., Choya-Foces C., Hugo M., López V. (2020). The Metabolite Urolithin-A Ameliorates Oxidative Stress in Neuro-2a Cells, Becoming a Potential Neuroprotective Agent. Antioxidants.

[138] Liu J., Jiang J., Qiu J., Wang L., Zhuo J., Wang B., Sun D., Yu S., Lou H. (2022). Urolithin A protects dopaminergic neurons in experimental models of Parkinson’s disease by promoting mitochondrial biogenesis through the SIRT1/PGC-1α signaling pathway. Food Funct..

[139] Kujawska M., Jourdes M., Kurpik M., Szulc M., Szaefer H., Chmielarz P., Kreiner G., Krajka-Kuźniak V., Mikołajczak P., Teissedre P.L. (2019). Neuroprotective Effects of Pomegranate Juice against Parkinson’s Disease and Presence of Ellagitannins-Derived Metabolite-Urolithin A-In the Brain. Int. J. Mol. Sci..

[140] Hazen S.L., Brown J.M. (2014). Eggs as a dietary source for gut microbial production of trimethylamine-N-oxide. Am. J. Clin. Nutr..

[141] Koeth R.A., Wang Z., Levison B.S., Buffa J.A., Org E., Sheehy B.T., Britt E.B., Fu X., Wu Y., Li L. (2013). Intestinal microbiota metabolism of L-carnitine, a nutrient in red meat, promotes atherosclerosis. Nat. Med..

[142] Jamal S., Kumari A., Singh A., Goyal S., Grover A. (2017). Conformational Ensembles of α-Synuclein Derived Peptide with Different Osmolytes from Temperature Replica Exchange Sampling. Front. Neurosci..

[143] Chung S.J., Rim J.H., Ji D., Lee S., Yoo H.S., Jung J.H., Baik K., Choi Y., Ye B.S., Sohn Y.H. (2021). Gut microbiota-derived metabolite trimethylamine N-oxide as a biomarker in early Parkinson’s disease. Nutrition.

[144] Stefanova N., Fellner L., Reindl M., Masliah E., Poewe W., Wenning G.K. (2011). Toll-like receptor 4 promotes α-synuclein clearance and survival of nigral dopaminergic neurons. Am. J. Pathol..

[145] Miranda A.F., Boegman R.J., Beninger R.J., Jhamandas K. (1997). Protection against quinolinic acid-mediated excitotoxicity in nigrostriatal dopaminergic neurons by endogenous kynurenic acid. Neuroscience.

[146] LeBlanc J.G., Milani C., de Giori G.S., Sesma F., van Sinderen D., Ventura M. (2013). Bacteria as vitamin suppliers to their host: A gut microbiota perspective. Curr. Opin. Biotechnol..

[147] da Silva F.L., Coelho Cerqueira E., de Freitas M.S., Gonçalves D.L., Costa L.T., Follmer C. (2013). Vitamins K interact with N-terminus α-synuclein and modulate the protein fibrillization in vitro. Exploring the interaction between quinones and α-synuclein. Neurochem. Int..

[148] Weller J., Budson A. (2018). Current understanding of Alzheimer’s disease diagnosis and treatment. F1000Res.

[149] Nisbet R.M., Götz J. (2018). Amyloid-β and Tau in Alzheimer’s Disease: Novel Pathomechanisms and Non-Pharmacological Treatment Strategies. J. Alzheimers Dis..

[150] Chen Y., Fang L., Chen S., Zhou H., Fan Y., Lin L., Li J., Xu J., Chen Y., Ma Y. (2020). Gut Microbiome Alterations Precede Cerebral Amyloidosis and Microglial Pathology in a Mouse Model of Alzheimer’s Disease. Biomed Res. Int..

[151] Gu X., Zhou J., Zhou Y., Wang H., Si N., Ren W., Zhao W., Fan X., Gao W., Wei X. (2021). Huanglian Jiedu decoction remodels the periphery microenvironment to inhibit Alzheimer’s disease progression based on the “brain-gut” axis through multiple integrated omics. Alzheimers Res. Ther..

[152] Emery D.C., Shoemark D.K., Batstone T.E., Waterfall C.M., Coghill J.A., Cerajewska T.L., Davies M., West N.X., Allen S.J. (2017). 16S rRNA Next Generation Sequencing Analysis Shows Bacteria in Alzheimer’s Post-Mortem Brain. Front. Aging Neurosci..

[153] Readhead B., Haure-Mirande J.V., Funk C.C., Richards M.A., Shannon P., Haroutunian V., Sano M., Liang W.S., Beckmann N.D., Price N.D. (2018). Multiscale Analysis of Independent Alzheimer’s Cohorts Finds Disruption of Molecular, Genetic, and Clinical Networks by Human Herpesvirus. Neuron.

[154] Zheng J., Zheng S.J., Cai W.J., Yu L., Yuan B.F., Feng Y.Q. (2019). Stable isotope labeling combined with liquid chromatography-tandem mass spectrometry for comprehensive analysis of short-chain fatty acids. Anal. Chim. Acta.

[155] Zhang L., Wang Y., Xiayu X., Shi C., Chen W., Song N., Fu X., Zhou R., Xu Y.F., Huang L. (2017). Altered Gut Microbiota in a Mouse Model of Alzheimer’s Disease. J. Alzheimers Dis..

[156] Park J., Kim C.H. (2021). Regulation of common neurological disorders by gut microbial metabolites. Exp. Mol. Med..

[157] Nagpal R., Neth B.J., Wang S., Craft S., Yadav H. (2019). Modified Mediterranean-ketogenic diet modulates gut microbiome and short-chain fatty acids in association with Alzheimer’s disease markers in subjects with mild cognitive impairment. EBioMedicine.

[158] Ferrante R.J., Kubilus J.K., Lee J., Ryu H., Beesen A., Zucker B., Smith K., Kowall N.W., Ratan R.R., Luthi-Carter R. (2003). Histone deacetylase inhibition by sodium butyrate chemotherapy ameliorates the neurodegenerative phenotype in Huntington’s disease mice. J. Neurosci..

[159] Ho L., Ono K., Tsuji M., Mazzola P., Singh R., Pasinetti G.M. (2018). Protective roles of intestinal microbiota derived short chain fatty acids in Alzheimer’s disease-type beta-amyloid neuropathological mechanisms. Expert. Rev. Neurother..

[160] Gong Z., Huang J., Xu B., Ou Z., Zhang L., Lin X., Ye X., Kong X., Long D., Sun X. (2019). Urolithin A attenuates memory impairment and neuroinflammation in APP/PS1 mice. J. Neuroinflamm..

[161] Ballesteros-Álvarez J., Nguyen W., Sivapatham R., Rane A., Andersen J.K. (2022). Urolithin A reduces amyloid-beta load and improves cognitive deficits uncorrelated with plaque burden in a mouse model of Alzheimer’s disease. Geroscience.

[162] Ali T., Kim T., Rehman S.U., Khan M.S., Amin F.U., Khan M., Ikram M., Kim M.O. (2018). Natural Dietary Supplementation of Anthocyanins via PI3K/Akt/Nrf2/HO-1 Pathways Mitigate Oxidative Stress, Neurodegeneration, and Memory Impairment in a Mouse Model of Alzheimer’s Disease. Mol. Neurobiol..

[163] Winter A.N., Bickford P.C. (2019). Anthocyanins and Their Metabolites as Therapeutic Agents for Neurodegenerative Disease. Antioxidants.

[164] Maitre M., Klein C., Patte-Mensah C., Mensah-Nyagan A.G. (2020). Tryptophan metabolites modify brain Aβ peptide degradation: A role in Alzheimer’s disease?. Prog. Neurobiol..

[165] Zhou H., Tai J., Xu H., Lu X., Meng D. (2019). Xanthoceraside Could Ameliorate Alzheimer’s Disease Symptoms of Rats by Affecting the Gut Microbiota Composition and Modulating the Endogenous Metabolite Levels. Front. Pharm..

[166] Drewes G., Ebneth A., Preuss U., Mandelkow E.M., Mandelkow E. (1997). MARK, a novel family of protein kinases that phosphorylate microtubule-associated proteins and trigger microtubule disruption. Cell.

[167] Matsumoto T., Nagase Y., Hirose J., Tokuyama N., Yasui T., Kadono Y., Ueki K., Kadowaki T., Nakamura K., Tanaka S. (2013). Regulation of bone resorption and sealing zone formation in osteoclasts occurs through protein kinase B-mediated microtubule stabilization. J. Bone Min. Res..

[168] Tseng H.C., Graves D.J. (1998). Natural methylamine osmolytes, trimethylamine N-oxide and betaine, increase tau-induced polymerization of microtubules. Biochem. Biophys. Res. Commun..

[169] Allison A.C. (2001). The possible role of vitamin K deficiency in the pathogenesis of Alzheimer’s disease and in augmenting brain damage associated with cardiovascular disease. Med. Hypotheses.

[170] Douaud G., Refsum H., de Jager C.A., Jacoby R., Nichols T.E., Smith S.M., Smith A.D. (2013). Preventing Alzheimer’s disease-related gray matter atrophy by B-vitamin treatment. Proc. Natl. Acad. Sci. USA.

[171] Walker F.O. (2007). Huntington’s disease. Lancet.

[172] Nayak A., Ansar R., Verma S.K., Bonifati D.M., Kishore U. (2011). Huntington’s Disease: An Immune Perspective. Neurol. Res. Int..

[173] Kong G., Ellul S., Narayana V.K., Kanojia K., Ha H.T.T., Li S., Renoir T., Cao K.L., Hannan A.J. (2021). An integrated metagenomics and metabolomics approach implicates the microbiota-gut-brain axis in the pathogenesis of Huntington’s disease. Neurobiol. Dis..

[174] Kong G., Cao K.L., Judd L.M., Li S., Renoir T., Hannan A.J. (2020). Microbiome profiling reveals gut dysbiosis in a transgenic mouse model of Huntington’s disease. Neurobiol. Dis..

[175] Du G., Dong W., Yang Q., Yu X., Ma J., Gu W., Huang Y. (2020). Altered Gut Microbiota Related to Inflammatory Responses in Patients With Huntington’s Disease. Front. Immunol..

[176] Stan T.L., Soylu-Kucharz R., Burleigh S., Prykhodko O., Cao L., Franke N., Sjögren M., Haikal C., Hållenius F., Björkqvist M. (2020). Increased intestinal permeability and gut dysbiosis in the R6/2 mouse model of Huntington’s disease. Sci. Rep..

[177] Stoy N., Mackay G.M., Forrest C.M., Christofides J., Egerton M., Stone T.W., Darlington L.G. (2005). Tryptophan metabolism and oxidative stress in patients with Huntington’s disease. J. Neurochem..

[178] Sun X., Zhu J., Sun X.Y., Ji M., Yu X.L., Liu R.T. (2020). Ellagic acid rescues motor and cognitive deficits in the R6/2 mouse model of Huntington’s disease by lowering mutant huntingtin protein. Food Funct..

[179] Rosen D.R., Siddique T., Patterson D., Figlewicz D.A., Sapp P., Hentati A., Donaldson D., Goto J., O’Regan J.P., Deng H.X. (1993). Mutations in Cu/Zn superoxide dismutase gene are associated with familial amyotrophic lateral sclerosis. Nature.

[180] Nicholson K., Bjornevik K., Abu-Ali G., Chan J., Cortese M., Dedi B., Jeon M., Xavier R., Huttenhower C., Ascherio A. (2021). The human gut microbiota in people with amyotrophic lateral sclerosis. Amyotroph Lateral Scler Front. Degener.

[181] Ngo S.T., Restuadi R., McCrae A.F., Van Eijk R.P., Garton F., Henderson R.D., Wray N.R., McCombe P.A., Steyn F.J. (2020). Progression and survival of patients with motor neuron disease relative to their fecal microbiota. Amyotroph Lateral Scler Front. Degener.

[182] Schwiertz A., Taras D., Schäfer K., Beijer S., Bos N.A., Donus C., Hardt P.D. (2010). Microbiota and SCFA in lean and overweight healthy subjects. Obesity.

[183] O’Reilly É.J., Wang M., Adami H.O., Alonso A., Bernstein L., van den Brandt P., Buring J., Daugherty S., Deapen D., Freedman D.M. (2018). Prediagnostic body size and risk of amyotrophic lateral sclerosis death in 10 studies. Amyotroph Lateral Scler Front. Degener.

[184] Paganoni S., Deng J., Jaffa M., Cudkowicz M.E., Wills A.M. (2011). Body mass index, not dyslipidemia, is an independent predictor of survival in amyotrophic lateral sclerosis. Muscle Nerve.

[185] Blacher E., Bashiardes S., Shapiro H., Rothschild D., Mor U., Dori-Bachash M., Kleimeyer C., Moresi C., Harnik Y., Zur M. (2019). Potential roles of gut microbiome and metabolites in modulating ALS in mice. Nature.

[186] Iłzecka J., Kocki T., Stelmasiak Z., Turski W.A. (2003). Endogenous protectant kynurenic acid in amyotrophic lateral sclerosis. Acta Neurol. Scand.

[187] Sospedra M., Martin R. (2005). Immunology of multiple sclerosis. Annu. Rev. Immunol..

[188] Chen J., Chia N., Kalari K.R., Yao J.Z., Novotna M., Paz Soldan M.M., Luckey D.H., Marietta E.V., Jeraldo P.R., Chen X. (2016). Multiple sclerosis patients have a distinct gut microbiota compared to healthy controls. Sci. Rep..

[189] Yokote H., Miyake S., Croxford J.L., Oki S., Mizusawa H., Yamamura T. (2008). NKT cell-dependent amelioration of a mouse model of multiple sclerosis by altering gut flora. Am. J. Pathol..

[190] Hirschberg S., Gisevius B., Duscha A., Haghikia A. (2019). Implications of Diet and The Gut Microbiome in Neuroinflammatory and Neurodegenerative Diseases. Int. J. Mol. Sci..

[191] Roager H.M., Licht T.R. (2018). Microbial tryptophan catabolites in health and disease. Nat. Commun..

[192] Jangi S., Gandhi R., Cox L.M., Li N., von Glehn F., Yan R., Patel B., Mazzola M.A., Liu S., Glanz B.L. (2016). Alterations of the human gut microbiome in multiple sclerosis. Nat. Commun..

[193] Gaetani L., Boscaro F., Pieraccini G., Calabresi P., Romani L., Di Filippo M., Zelante T. (2020). Host and Microbial Tryptophan Metabolic Profiling in Multiple Sclerosis. Front. Immunol..

[194] Nourbakhsh B., Bhargava P., Tremlett H., Hart J., Graves J., Waubant E. (2018). Altered tryptophan metabolism is associated with pediatric multiple sclerosis risk and course. Ann. Clin. Transl. Neurol..

[195] Lieben C.K., Blokland A., Deutz N.E., Jansen W., Han G., Hupperts R.M. (2018). Intake of tryptophan-enriched whey protein acutely enhances recall of positive loaded words in patients with multiple sclerosis. Clin. Nutr..

[196] Rothhammer V., Mascanfroni I.D., Bunse L., Takenaka M.C., Kenison J.E., Mayo L., Chao C.C., Patel B., Yan R., Blain M. (2016). Type I interferons and microbial metabolites of tryptophan modulate astrocyte activity and central nervous system inflammation via the aryl hydrocarbon receptor. Nat. Med..

[197] Rothhammer V., Borucki D.M., Tjon E.C., Takenaka M.C., Chao C.C., Ardura-Fabregat A., de Lima K.A., Gutiérrez-Vázquez C., Hewson P., Staszewski O. (2018). Microglial control of astrocytes in response to microbial metabolites. Nature.

[198] Bhargava P., Smith M.D., Mische L., Harrington E., Fitzgerald K.C., Martin K., Kim S., Reyes A.A., Gonzalez-Cardona J., Volsko C. (2020). Bile acid metabolism is altered in multiple sclerosis and supplementation ameliorates neuroinflammation. J. Clin. Investig..

[199] Sarkar A., Lehto S.M., Harty S., Dinan T.G., Cryan J.F., Burnet P.W.J. (2016). Psychobiotics and the Manipulation of Bacteria–Gut–Brain Signals. Trends Neurosci..

[200] Zhou L., Foster J.A. (2015). Psychobiotics and the gut-brain axis: In the pursuit of happiness. Neuropsychiatr. Dis. Treat..

[201] Barrio C., Arias-Sánchez S., Martín-Monzón I. (2022). The gut microbiota-brain axis, psychobiotics and its influence on brain and behaviour: A systematic review. Psychoneuroendocrinology.

[202] Mayorgas A., Dotti I., Salas A. (2021). Microbial Metabolites, Postbiotics, and Intestinal Epithelial Function. Mol. Nutr. Food Res..

[203] Żółkiewicz J., Marzec A., Ruszczyński M., Feleszko W. (2020). Postbiotics—A Step Beyond Pre- and Probiotics. Nutrients.

[204] Irving A.T., Mimuro H., Kufer T.A., Lo C., Wheeler R., Turner L.J., Thomas B.J., Malosse C., Gantier M.P., Casillas L.N. (2014). The Immune Receptor NOD1 and Kinase RIP2 Interact with Bacterial Peptidoglycan on Early Endosomes to Promote Autophagy and Inflammatory Signaling. Cell Host Microbe.

[205] Dinić M., Lukić J., Djokić J., Milenković M., Strahinić I., Golić N., Begović J. (2017). Lactobacillus fermentum Postbiotic-induced Autophagy as Potential Approach for Treatment of Acetaminophen Hepatotoxicity. Front. Microbiol..

[206] Shen T.D. (2017). Diet and Gut Microbiota in Health and Disease. Nestle Nutr. Inst. Workshop Ser..

[207] Bagherniya M., Butler A.E., Barreto G.E., Sahebkar A. (2018). The effect of fasting or calorie restriction on autophagy induction: A review of the literature. Ageing Res. Rev..

[208] Zhang Y., Liu C., Zhao Y., Zhang X., Li B., Cui R. (2015). The Effects of Calorie Restriction in Depression and Potential Mechanisms. Curr. Neuropharmacol..

[209] Ghosh T.S., Rampelli S., Jeffery I.B., Santoro A., Neto M., Capri M., Giampieri E., Jennings A., Candela M., Turroni S. (2020). Mediterranean diet intervention alters the gut microbiome in older people reducing frailty and improving health status: The NU-AGE 1-year dietary intervention across five European countries. Gut.

[210] Staley C., Khoruts A., Sadowsky M.J. (2017). Contemporary Applications of Fecal Microbiota Transplantation to Treat Intestinal Diseases in Humans. Arch. Med. Res..

[211] Donohoe D.R., Garge N., Zhang X., Sun W., O’Connell T.M., Bunger M.K., Bultman S.J. (2011). The Microbiome and Butyrate Regulate Energy Metabolism and Autophagy in the Mammalian Colon. Cell Metab..

[212] Lai C.H., Tsai C.C., Kuo W.W., Ho T.J., Day C.H., Pai P.Y., Chung L.C., Huang C.C., Wang H.F., Liao P.H. (2016). Multi-Strain Probiotics Inhibit Cardiac Myopathies and Autophagy to Prevent Heart Injury in High-Fat Diet-Fed Rats. Int. J. Med. Sci..

[213] Qi R., Sun J., Qiu X., Zhang Y., Wang J., Wang Q., Huang J., Ge L., Liu Z. (2021). The intestinal microbiota contributes to the growth and physiological state of muscle tissue in piglets. Sci. Rep..

